# Use of Systemic Steroids, Hormone Replacement Therapy, or Oral Contraceptives Is Associated with Decreased Implant Survival in Women

**DOI:** 10.3390/dj11070163

**Published:** 2023-06-29

**Authors:** Michelle Y. Zou, Robert E. Cohen, Brendon L. Ursomanno, Lisa M. Yerke

**Affiliations:** Department of Periodontics and Endodontics, School of Dental Medicine, University at Buffalo, The State University of New York, Buffalo, NY 14214, USA; michellezou324@gmail.com (M.Y.Z.);

**Keywords:** dental implants, implant failure, steroids, birth control, hormone replacement therapy

## Abstract

Background: Systemic steroids, such as prednisone, hormonal replacement therapies, or oral contraceptives, are commonly prescribed to women who might also be receiving dental implant therapy. However, the effect of these medications on dental implant survival is unknown. Methods: The medical and dental records of individuals with dental implants (N = 1480 implants) who visited a postgraduate periodontics clinic between 2000 and 2017 were initially considered. Those younger than 21 years old, pregnant, or male were excluded according to the study’s exclusion criteria. The presence of systemic diseases and conditions was assessed. Implant failure rates among female patients using systemic steroids, hormone replacement therapy, or oral contraceptives were compared with failure rates among patients not taking those medications. Results: The implant failure rate for the 65 implants in patients taking steroid medications was 7.69%; the failure rate for the 712 implants in patients not taking steroids was 1.54% (*p* < 0.001). After adjusting for smoking and the presence of diabetes, that relationship persisted, with an 8.47% implant failure rate for the 59 implants in patients taking steroids (vs. 1.54% failure for the 585 implants in patients not taking steroids; *p* < 0.001). Regression analyses demonstrated that the odds of implant failure versus success were 5.31 times greater in patients taking systemic steroids, hormone replacement therapy, or oral contraceptives (*p* < 0.05). No statistically significant differences in patient plaque control were found between the experimental and control groups. Conclusions: Among women, the use of systemic steroids is associated with a five-fold increase in the rate of dental implant failure, regardless of the presence of smoking or diabetes.

## 1. Introduction

Corticosteroid medications are among the most frequently prescribed medications worldwide, having an annual worldwide market value of approximately USD 10 billion [1]. Corticosteroids have anti-inflammatory and immunosuppressive properties and are used to manage a variety of systemic conditions, such as rheumatoid arthritis and allergies [2]. In dentistry, corticosteroids can be used for the treatment of pemphigus, pemphigoid, lichen planus, and recurrent aphthous stomatitis, as well as to minimize postoperative swelling [3]. Corticosteroids inhibit fibroblast proliferation, reduce collagen formation, and can induce alterations in bone cell subpopulations [4]. They affect the metabolic activity of osteoblasts and osteoclasts, promote apoptosis in osteoblasts and osteocytes, and prolong the lifespan of osteoclasts. Collectively, those effects can result in decreased bone formation and increased bone resorption, resulting in bone loss [5]. 

Corticosteroids function by binding to nuclear receptors and, once activated, can affect cell proliferation, development, metabolism, and reproduction [6]. They bind to transport proteins within the circulatory system and undergo conformational change during ligand binding. Corticosteroids then dissociate from their transport proteins and diffuse through the capillary wall and cellular membranes of target cells [7]. Once the corticosteroids have crossed the membrane and entered the nucleus of target cells, they bind directly to glucocorticoid response elements that consist of specific DNA sequences. This hormone–DNA complex stimulates target gene expression to enhance or repress gene transcription and subsequently influence a variety of physiologic processes [1,8].

Systemic steroids are an integral component of hormone replacement therapy (HRT) and oral contraceptives (OC). HRT is generally characterized by the administration of sex hormones as a strategy to manage insufficient hormone levels, particularly for women experiencing menopausal symptoms. Conventional HRT consists of systemically administered estrogen and progesterone [9]. On the other hand, OC are prescribed to prevent pregnancy and typically consist of either a combined formulation of estrogen-progesterone or of only progesterone [10]. Progesterone and estrogen suppress both follicle-stimulating hormone and luteinizing hormone to prevent ovulation [11]. The most common side effects of HRT and OC include weight gain, nausea, swelling, dizziness, and headaches [12,13].

Studies have suggested that HRT and OC might have adverse effects on the periodontium [4,14,15]. Supplementation with systemic steroids initiates immunological changes, leading to alterations in fibroblast proliferation, collagen production, and bone regeneration, as well as increased periodontal probing depths. Progressive periodontitis has been associated with the prolonged use of systemic steroids [4]. Several clinical studies have shown that patients taking OC exhibit greater concentrations of C-reactive protein (an indicator of inflammation and tissue damage) [15], more pronounced gingival inflammation, increased loss of clinical attachment, and gingival enlargement [14]. Similarly, studies describing the effects of pregnancy on the periodontium have shown increased gingival inflammation and probing depths at the second month of pregnancy, reaching a maximum at 8 months. The prevalence and severity of gingival disease typically declines post-partum [16]. Additionally, receptors for estrogen and progesterone can be identified in the gingiva, periosteum, and periodontal ligament fibroblasts and osteoblasts. Estrogen also can influence the permeability of the oral epithelium to bacteria and can affect collagen repair and maintenance, while progesterone can upregulate the production of inflammatory mediators such as prostaglandins and polymorphonuclear leukocytes [17]. The identification of those receptors suggests that steroids can affect the physiology and inflammatory responses of oral tissues [18]. 

The use of dental implants is an effective method for the replacement of missing teeth, with approximately 100,000–300,000 dental implants placed annually [19]. The long-term success of implant dentistry depends primarily on osseointegration, the fusion between the bone and the implant surface that prevents movement or fracture of the prosthesis. Dental implants have the highest predictability of success when there is adequate quantity and quality of bone to allow osseointegration [20]. The outcome of implant therapy can be assessed by several clinical measurements, including the presence of pain, mobility, crestal bone loss, probing depth, and lack of peri-implantitis. 

A large-scale meta-analysis of 13,049 two-stage implants showed a survival rate of 92% over 15 years, and 85% for 5515 one-stage surgery implants over 10 years [21]. Implant complications include peri-implantitis, infection, implant breakage, and pain [22]. Such complications can increase the risk of implant failure. Smoking [23], some systemic conditions such as diabetes, belonging to the male gender, and poor oral hygiene are all additional risk factors for implant failure [24]. Similarly, the use of systemic steroidal medications has been shown to have a negative association with the periodontium, increasing the risk for periodontitis and periodontopathogens [25]. The use of other medications, such as non-steroidal anti-inflammatory drugs and selective serotonin reuptake inhibitors, have been shown to impact dental implant failure [26]; however, the influence of systemic steroids on dental implants has not been completely elucidated. As a result, the first aim of the present study was to measure the rate of dental implant failure among women taking systemic steroids, HRT, or OC and to compare those findings to patients not taking those medications. The second aim was similar; however, it excluded women taking systemic steroid medications for chronic diseases or other medical conditions and was intended to measure the rate of dental implant failure among women taking either hormone replacement therapy or oral contraceptives and compare those findings to patients not taking those medications. We hypothesized that dental implants in patients taking steroids/HRT/OC (aim 1) or patients taking HRT/OC (aim 2) experience a greater rate of dental implant failure than those not taking those medications.

## 2. Materials and Methods

### 2.1. Patient Population

This project was reviewed and approved by the University at Buffalo Health Sciences Institutional Review Board (IRB) (STUDY00002276). Due to the retrospective nature of the study, the University at Buffalo Health Sciences IRB waived the need for informed consent. The manuscript was prepared according to the Strengthening the Reporting of Observational Studies in Epidemiology (STROBE) guidelines [27]. For this retrospective clinical study, all methods were performed in accordance with the relevant guidelines and regulations. Female patients aged 21 years or older who had received implant therapy at the University at Buffalo School of Dental Medicine Postgraduate Periodontics Clinic from 2000 to 2017 were considered (N = 777 implants). For each patient, medical and dental histories were obtained, including smoking status, presence of diabetes, and use of medications, including systemic steroids (including but not exclusive to prednisone) or use of HRT or OC. Patients without either diagnostic radiographs or clinical evaluation at the time of both implant placement and subsequent reevaluation were excluded. Patients with autoimmune conditions or taking medications such as immunomodulators also were excluded. Radiographic analysis was conducted using film images or via the use of MIPACS Dental Enterprise Viewer software 3.0 for digital images (Medicor Imaging; LEAD Technologies, Inc.; Charlotte, NC, USA). Periapical and panoramic radiographs and patient histories were reviewed to assess whether implant osseointegration was obtained following implant placement and again referenced at dates following osseointegration during the study period to determine if the implant was removed or lost.

### 2.2. Criteria Analysis

Radiographs were reviewed by a single examiner (B.U.). A retrospective review of patient charts was performed to evaluate implants at the time of initial placement as well as at subsequent reevaluation to identify patients with failed implants (i.e., loss of osseointegration to an extent where removal was required or implants that no longer were present). To eliminate the potentially confounding effects of smoking and diabetes on implant failure, the analysis was repeated after excluding such patients.

### 2.3. Statistical Analysis

For analysis, Fisher’s exact test was used to compare implant failure vs. success among patients either taking systemic steroids/HRT/OC or HRT/OC with those not taking those medications. To determine whether the difference in each patient’s oral hygiene had an impact on implant success, each patient’s plaque score was obtained using the Ramfjord index sampling technique [16] or via whole-mouth plaque detection [28], and mean values (percentage of teeth with plaque accumulation) were calculated. Independent t-tests were used to assess plaque index and age among groups, with significance measured at *p* < 0.05 for group mean differences. The percentage of implant failure was determined by dividing the number of failed implants by the total number of implants placed in each group. Backward selection using a significance level of 0.05 was performed using implant failure as an outcome and medication use, presence of diabetes, and smoking status as predictors to choose a model for regression analyses. Regression analyses were performed using SAS 9.4 (IBM, Cary, NC, USA); all other statistical calculations were performed using SPSS Statistics v26 (IBM, Armonk, NY, USA).

## 3. Results

The failure rate was 7.69% for the 65 implants in patients (mean age 61.1, SD = 15.3) taking any systemic steroid medication (systemic steroids/HRT/OC) compared to 1.54% for the 712 implants in patients (mean age 62.8, SD = 16.1) not taking steroids (*p* < 0.001). That relationship persisted after excluding patients who were smokers or patients with diabetes, with an implant failure rate of 8.49% for the 59 implants in patients (mean age 61.0, SD = 16.0) taking systemic steroids, compared to 1.54% for the 585 implants in patients (mean age 64.2, SD = 16.9) not taking those medications (*p* < 0.001). There were no statistically significant differences in age among the individuals taking or not taking steroid medications (*p* < 0.05). These findings demonstrate an approximately five-fold increase in implant failure among patients taking steroids compared to patients not taking those medications and were independent of the presence of smoking or diabetes. These results are summarized in Table 1.

When systemic steroid medications prescribed for the treatment of chronic diseases and other conditions were excluded from the analysis, meaning only women taking hormone replacement therapy or oral contraceptive were considered, the implant failure rate was 8.62% for the 58 implants in patients (mean age 60.1, SD = 16.1) taking HRT/OC medications and to 1.53% for the 719 implants in patients (mean age 62.9, SD = 16.1) not taking HRT/OC medications (*p* = 0.005). That relationship persisted after excluding patients who were smokers or those with diabetes (*p* = 0.003; Table 2). These findings also suggest a more than five-fold increase in dental implant failure for women taking oral contraceptives or hormone replacement therapy compared to women not taking those medications. Similarly, there were no statistically significant differences in age among the individuals taking or not taking steroid medications (*p* > 0.05).

Backward selection was used to determine an appropriate model for regression analysis. The presence of diabetes and smoking were thereby removed as non-significant variables, while systemic steroids, hormone replacement therapy, and oral contraceptive steroid medications were included, as well as when only HRT/OC medications were included. The odds of implant failure (relative to implant success) were 5.31 times more likely among patients taking steroids/HRT/OC (CI: 1.79–15.79, *p* < 0.05). Similarly, the odds of implant failure were 6.07 more likely among patients taking HRT/OC (CI: 2.04–18.12, *p* < 0.05).

Finally, no statistically significant differences in patient plaque control were found between patients taking steroids vs. not taking steroids, suggesting that difference in patient plaque control was not a confounding factor that could affect the outcome.

## 4. Discussion

The results of the present study suggest that steroid medications are related to increased implant failure. This relationship persisted regardless of whether steroids were classified as systemic steroid medications/HRT/OC or only as HRT/OC. In addition, these findings appear to be free from the influence of smoking, diabetes, the use of immunobiologic medications, or the presence of systemic immunologic conditions or diseases. Previous studies have shown an association between steroids, bone resorption, and periodontitis. Corticosteroids stimulate osteoclastogenesis, which leads to increased bone resorption and the induction of inflammatory mediators in the periodontium. Several clinical studies have reported that corticosteroids are associated with alterations in the development and progression of periodontal disease, such as impaired bone metabolism and increased presence of caries, plaque, calculus, and *Candida* species [4]. 

Other clinical studies have provided insight about the mechanism in which glucocorticoids might affect bone [29,30,31]. One study [30] showed that treatment with short-term glucocorticoids resulted in a rapid and significant decrease in type I procollagen N-terminal propeptide and osteocalcin, which are markers for bone formation. There was also a decrease in IGF2, a regulator of osteoblast function, and an increase in the expression of the RANK ligand, with a decrease in the expression of osteoprotegerin in osteoblastic and stromal cells. Osteoprotegerin functions as a decoy receptor that opposes the RANK ligand is a cytokine receptor that activates osteoclasts and causes bone resorption [32]. These changes suggested that glucocorticoids play a role in modulating the bone cell subpopulation. When the intake of glucocorticoids was discontinued during a two-week recovery period, these changes were reversed. The aforementioned study demonstrated that elevated concentrations of glucocorticoids might decrease the rate of bone formation, as well as the number and activity of osteoblasts and osteocytes, with an increase in osteoclast lifespan [30]. Another study [29] in mice demonstrated that the long-term use of glucocorticoids is associated with significantly decreased alveolar bone thickness and volume, as well as decreased bone formation with reduced mineral apposition rate. This finding is consistent with a study by Chen et al. [31], who showed that glucocorticoid treatment in rats for 21 days decreased bone mineral density in the femur. Collectively, the data support that glucocorticoids alter inflammatory pathways that drive bone formation and resorption.

A retrospective study by August et al. [33] found that postmenopausal women without estrogen replacement therapy (ERT) experienced more implant failure in the maxilla compared to those with ERT. Comparatively, in the mandible, postmenopausal women with ERT experienced more implant failure than those with ERT. However, both findings were not significant. It was suggested that statistical significance was not achieved (*p* = 0.17) due to the small sample size and low statistical power, and in order to detect a difference with 80% power and 0.05 significance, the number of implants in each group would need to be increased to 536. It was also speculated that the cause could be due to the fewer number of implants in groups with ERT compared to those without ERT. In the current study, each group had a larger sample size of female participants receiving implant therapy, and the results were statistically significant (*p* < 0.001). 

A study by Minsk and Polson [34] suggested that hormone replacement therapy is not associated with improved dental implant treatment in postmenopausal women. In a study by Moy et al. [35], patients who were on hormone replacement therapy experienced significantly increased implant failure compared to healthy patients. Additionally, postmenopausal estrogen was correlated with a significantly increased failure rate, with a relative risk of 2.55. Relative risk ratios were calculated using correlations between data on gender, age, implant location, bone quality, bone volume, and medical history, and implant failure. Both Moy et al. and Minsk and Polson’s studies evaluated only postmenopausal women when observing the effect of ERT on implant failure, and both the medication dose and the duration of treatment were unclear. Moreover, postmenopausal women are more susceptible to osteoporosis primarily due to estrogen deficiency, which can influence the results of their study [36]. Koszuta et al. found that HRT significantly contributes to greater peri-implant bone loss [37]. In Tounta’s cross-sectional study, he concluded that glucocorticoids decreased calcium levels, resulting in an increased risk of decreased bone mineralization. Tounta’s study suggested that patients who take systemic steroid medications up to 0.5mg/kg/d were at risk of worsening bone quality and osteoporosis [38]. Another retrospective study showed that the use of chronic glucocorticoid was associated with a 20% implant failure rate [39]. Collectively, these results are consistent with the results of the present study. 

The inconsistent findings for human subjects reported in the literature [33,34,35] show that further studies into the influence of systemic steroidal medication on implant success is required. Additionally, those studies, as well as a systemic review [33,34,35,40], included patients with systemic diseases and did not exclude subjects based on other factors besides medications that may cause increased implant failure. 

Conversely, Qi et al. [41] described an animal study that correlated ERT with bone healing around titanium implants. However, that study observed bone level around implants placed in the tibial metaphysis of rats. That study also was limited by a small sample size (N = 60), with 6 of the rats being sacrificed after ovariectomy. Additionally, estradiol levels before implantation and before estrogen therapy were not quantified, and their results found new bone in contact with the implant within cortical bone in all study groups. This suggests that rats without ERT are still able to undergo bone healing, but it might occur at a slower rate. Further investigations directed toward human studies with implants placed in alveolar bone would provide further insight into the direct impact of estrogen therapy on dental implant osseointegration [41]. 

The limitations of our study include an implant assessment period of 17 years, in which implants were placed by multiple surgeons, thereby possibly introducing some inter-clinician variability. Implant location and type were not analyzed, and there was a lack of information regarding the extent of time each patient was taking steroids, the medication dosage, and the temporal relationship between steroid use and implant placement, which is characteristic of retrospective studies in general. Therefore, it is not possible to determine if the association was due to the steroid medication or due to the condition that necessitated medication use. However, since major systemic diseases were part of the exclusion criteria, the majority of study subjects were healthy women, minimizing the effects of this bias. Finally, in a second analysis to further address this limitation, we eliminated patients taking steroids to treat systemic conditions other than for hormonal replacement purposes or birth control, and this did not change the significance of our results. Potential confounders such as diabetes and smoking were also removed from our multivariate regression analysis due to their non-significance in the model. Steroids/HRT/OC or HRT/OC remained significantly associated with implant failure, even when patients who smoked or had diabetes were excluded from the regression analysis, further supporting the association between medication and implant failure.

The current study demonstrates that patients taking systemic steroids/HRT/OC or HRT/OC medications are at a significantly greater risk of experiencing implant failure compared to patients not taking those medications. The results were similar when the entire population was assessed vs. when diabetics and smokers and patients with systemic conditions were excluded. The findings suggest that steroidal medications might impact implant failure, regardless of systemic diseases or whether the patient is a smoker or has diabetes. However, prospective clinical trials are indicated to determine if steroid medications can cause implant failure or if the relationship is only associative. Since we observed a five-fold increase in the rate of implant failure when patients were taking systemic steroids/HRT/OC, our findings suggest that clinicians might consider the clinical significance of steroid use as a potential risk factor for implant failure among patients taking those medications.

## 5. Conclusions

The results of this study support the hypothesis that the use of steroid medication is associated with dental implant failure among women, regardless of systemic diseases or whether the patient is a smoker or has diabetes. Although a history of steroid use does not appear to be a contraindication to implant therapy, clinicians nevertheless should consider a patient’s medication history when assessing the appropriateness of implant placement and during the informed consent process if appropriate.

## Figures and Tables

**Table 1:** Implant failure rates: systemic steroids, hormone replacement therapy, and oral contraceptive steroid medications all included.

Patient Characteristics	All Patients	Excluding Smoking and Patients with Diabetes
Failure rate among women taking steroids ¹^,2^ (%)	7.69 (N ^3^ = 65)	8.47 (N = 59)
Failure rate among women not taking steroids ¹^,2^ (%)	1.54 (N = 712)	1.54 (N = 585)
*p*-value Fisher’s exact degrees of freedom Failure/success odds ratio	*p* < 0.001 X^2^ = 11,161 df = 1 0.188	*p* < 0.005 X^2^ = 12,124 df = 1 0.169

^1^ “Steroids” include systemic medications, hormone replacement therapy, and oral contraceptives. ^2^ No statistical significance was detected in plaque control or age among women taking steroids or those not taking steroids. ^3^ N equals the total number of dental implants among women in that steroid group.

**Table 2:** Implant failure rates: hormone replacement therapy or oral contraceptive steroid medications only.

Patient Characteristics	All Patients	Excluding Smoking and Patients with Diabetes
Failure rate among women taking steroids ^1,2^ (%)	8.62 (N ^3^ = 58)	9.62 (N = 52)
Failure rate among women not taking steroids ^1,2^ (%)	1.53 (N = 719)	1.52 (N = 592)
*p*-value Fisher’s exact degrees of freedom Failure/success odds ratio	*p* = 0.005 X^2^ = 13,380 df = 1 0.021	*p* = 0.003 X^2^ = 14,730 df = 1 0.022

^1^ “Steroids” include hormone replacement therapy and oral contraceptives only. ^2^ No statistical significance was detected in plaque control or age among women taking steroids or those not taking steroid. ^3^ N equals the total number of dental implants among women in that steroid group.

## Data Availability

The dataset analyzed in this study is available from the corresponding author on reasonable request.
